# Production, Characterization, and Antimicrobial Activity of Mycocin Produced by* Debaryomyces hansenii* DSMZ70238

**DOI:** 10.1155/2017/2605382

**Published:** 2017-07-03

**Authors:** Safaa A. S. Al-Qaysi, Halah Al-Haideri, Zaid Akram Thabit, Wijdan Hameed Abd Al-Razzaq Al-Kubaisy, Jamal Abd Al-Rahman Ibrahim

**Affiliations:** ^1^Department of Biology, College of Science for Women, University of Baghdad, Baghdad, Iraq; ^2^Environmental Biotechnology Department, Biotechnology Research Center, Al Nahrain University, Baghdad, Iraq; ^3^Department of Biology, College of Education for Pure Science, Al-Anbar University, Ramadi, Iraq

## Abstract

The present study was conducted to estimate the antimicrobial activity and the potential biological control of the killer toxin produced by* D. hansenii* DSMZ70238 against several pathogenic microorganisms. In this study, the effects of NaCl, pH, and temperature, killer toxin production, and antimicrobial activity were studied. The results showed that the optimum inhibitory effect of killer toxin was at 8% NaCl, and the diameters of clear zones were 20, 22, 22, 21, 14, and 13 mm for* Staphylococcus aureus, Escherichia coli, Klebsiella pneumoniae, Streptococcus pyogenes, Candida albicans, *and* Candida neoformans*, respectively. The largest inhibition zones were observed at pH 4.5 with inhibition zone of 16, 18, 17, 18, 11, and 12 mm for the same microorganisms. The results also showed that 25°C is the optimal temperature for toxin killing activity against all targeted microorganisms. In addition, the activity of killer toxin significantly inhibited the growth of fungal mycelia for all target pathogenic fungi and the percentages of inhibition were 47.77, 48.88, 52.22, and 61.11% for* Trichophyton rubrum, Alternaria alternata, Trichophyton concentricum, *and* Curvularia lunata*, respectively. The results showed the highest growth rate of* D. hansenii* DSMZ70238 under condition of 8% NaCl concentration, pH 4.5, and 25°C for 72 h.

## 1. Introduction

*Debaryomyces hansenii* is one of the most common yeast species in nature and food such as dairy products, including soft, brine, and hard cheese [1–3].* D. hansenii* is a halotolerant species and it can be grown in medium supplemented with up to 25% sodium chloride [4]. In addition, it can survive at pH levels ranging between 3 and 10 and low levels of water activity [5]. It has a halo-xerotolerance trait and shows a broad spectrum of carbon source assimilation and fermentation [6]. The genus* Debaryomyces *Lodder et Kreger-van Rij is a genus of yeasts that belongs to the family of Saccharomycetaceae. In general, most of* D. hansenii* strains are haploid and can be reproduced vegetatively by forming of multilateral budding [3] via sexual reproduction (heterogamous conjugation) leading to short diplophase and then by meiosis and ascospore formation. These yeasts are distributed as commensals in the healthy persons but can be pathogenic microorganisms and cause a disease called candidemia after disturbance occurs in the host defense barriers [7, 8]. Many species of yeast have been reported to produce toxic proteins called (exotoxin), also named mycocins, against several pathogenic yeasts such as* Candida albicans*.* D. hansenii* has been reported to produce strong and active toxic proteins or glycoproteins, as killer toxins. These antimicrobial substances can play an important role in the inhibition growth of different genera of yeasts. The production of killer toxin was firstly described in* Saccharomyces cerevisiae* in 1963; after that the activity of killer toxin has been reported in 100 yeast species belonging to more than twenty genera [9]. Mode of action of these mycocins may be belonging to several mechanisms that involve DNA damage and inhibition of DNA replication, attacking the cell membranes of target yeast cells and pores formation. This can lead to changes in the permeability of the cytoplasmic membrane and loss of potassium ions and energy in the form of ATP or arrest the cell cycle of yeast cells at the G1 phase [10–12].

Killer toxins produced by* D. hansenii* showed antimicrobial efficiency towards several yeast species, especially the opportunistic pathogenic* Candida* spp. [13, 14].* D. hansenii* was used as a biological control agent against some molds such as* Penicillium digitatum* and* P. italicum* [6, 15] and phytopathogenic fungi such as* Mucor circinelloides*,* Fusarium proliferatum, *and* F. subglutinans* that infect* Zea mays L. grains* [16]. It was also used as a bioprotective agent against some dairy molds including* Aspergillus *sp.*, Byssochlamys fulva, B. nivea, Cladosporium *sp.*, Eurotium chevalieri, P. candidum, *and* P. roqueforti *[17]. However, studies in the field of the antibacterial activity of mycotoxin produced by* D. hansenii* against Gram positive and negative bacteria are very rare. One of these studies [18] evaluated the antimicrobial activity of* D. hansenii* toward* Clostridium tyrobutyricum *and* C. butyricum*. In this work, the main objectives are production, detection, and testing of the killer activity of mycocin produced by* D. hansenii* DSMZ70238 against several types of microorganisms, including pathogenic yeasts, bacteria, and fungi.

## 2. Materials and Methods

### 2.1. Microorganisms and Its Maintenance

*D. hansenii* DSMZ70238 strain was obtained from the German Collection of Microorganisms and Cell cultures (DSMZ) and used to produce the killer toxin. Yeast cells were grown and maintained in Yeast Extract-Peptone Dextrose (YEPD) agar slants medium containing 2.0% glucose, 2.0% peptone, 1.0% yeast extract, and 2.0% agar at 4°C. Pathogenic strains of* Candida albicans, Cryptococcus neoformans, Staphylococcus aureus, Escherichia coli, Klebsiella pneumonia, Streptococcus pyogenes, Trichophyton rubrum*,* Trichophyton concentricum, Alternaria alternata, *and* Curvularia lunata * were used. These microorganisms were obtained from laboratories of microbiology, Department of Biology, Faculty of Science For Women, University of Baghdad. They were originally isolated from clinical samples. Identification and characterization were done according to biochemical, morphological, and physiological tests. All isolates were subcultured and maintained on selective media for further use.

### 2.2. Production of Killer Toxin

*D. hansenii* DSMZ70238 was grown for 72 h at 25°C in 250 ml Erlenmeyer flasks with 100 mL of the YEPD broth medium at pH 4.5. The cultures were aerobically incubated with gentle shaking (130 rpm). The biomass was separated from the supernatant by centrifugation at 5000 ×g for 10 min at 4°C. The free cell supernatant was adjusted to a final glycerol concentration of 15% and concentrated to a volume of 15 mL by using ultrafiltration with appropriate device size and membrane (Vivaspin®6, 5-kDa-cutoff membrane PES, Sartorius, Germany). This partially purified concentrated supernatant was used as a concentrated killer toxin after sterilization through a 0.45 *µ*m pore-size membrane Millipore filters [19].

### 2.3. One-Dimensional SDS-Polyacrylamide Gel Electrophoresis (SDS-PAGE)

SDS-PAGE was performed in order to identify the produced killer toxins. A 12% resolving gel was prepared by adding 30% polyacrylamide, 10% [w/v] SDS, Tris-HCl pH 8.8, 10% [w/v] ammonium persulfate (APS) N,N,N,N tetramethylethylenediamine (TEMED), and water. The mixture was poured into the clean glass and allowed to settle. After gel assemble, a 6% stacking gel was prepared from the same components but with Tris-HCl pH 6.8 instead of Tris-HCl pH 8.8. The mixture was poured above the resolving gel with 1.0 mm comb and left to solidify. The produced killer toxin was prepared by mixing with 6x SDS loading dye (0.375 M [w/v] Tris-HCl pH 6.8, 2 g [v/v] glycerol, 4 g [w/v] SDS, 0.02 g [w/v] bromophenol blue, and adequate amount of B-mercaptoethanol) and then boiling at 95°C for 5 min. The comb was removed and the gel was located in a gel tank with 1x SDS running buffer (25 mM Tris, 250 mM glycine, and 0.1% [w/v] SDS). The samples were then loaded into the gel with unstained protein ladder (Promega, USA). The gel was electrophoresed at a constant 180 v until the dye reached the bottom or as desired. Gels were stained with Coomassie blue 50% [v/v] methanol, 10% [v/v] glacial acetic acid, and 0.1% [w/v] Coomassie brilliant blue R (Sigma-Aldrich) and destined in 30% [v/v] methanol and 10% [v/v] glacial acetic acid twice or until the proteins became visible.

### 2.4. Antifungal and Antibacterial Activity of Killer Toxin

The killer toxin activity of* D. hansenii* toward sensitive yeast strains was assessed by using the agar diffusion well method [20]. YEPD-methylene blue agar was prepared by adding 0.003% methylene blue to the YEPD agar medium dissolved in the 10 mM citrate/phosphate buffer and pH 4.5 for yeast strains. The medium was seeded with sensitive yeast* (C. albicans, C. neoformans)* at a final density of 1 × 10^5^ cells ml/mL before being poured. Wells were cut in the medium using a sterile cork borer (5 mm diameter) and agar plugs removed with a sterile scalpel. Finally, 100 *µ*L of concentrated killer toxin extract was added to each well and incubated at 25°C for 48 h. The existence of a halo zone around the wells was recorded and measured. The assay was performed in triplicate [19, 21].

The cells of pathogenic bacteria were grown in nutrient broth for 24 h with gentle shaking (120 rpm) at 37°C, and a 100 *µ*l of 1 × 10^5^ cells was spread out on Muller Hinton Agar. Wells in the size of 5 mm were cut by using sterile cork borer, and up to 100 *µ*l of concentrated killer toxin extract was added to each well. The plates were incubated at 37°C for 24–48 h and the diameter of the clear zone around the wells was measured [22]. The assay was repeated three times from three independent biological replicates.

### 2.5. Antagonistic Activity of* D. hansenii* against Pathogenic Fungi

The antagonistic effect of* D. hansenii* was evaluated on four pathogenic fungi strains, including two strains of human pathogens (*T. rubrum* and* T. concentricum*) and two plant pathogens (*A. alternata* and* C. lunata*). The activity of* D. hansenii* was performed by agar plate inhibition assay, as described by Núñez et al. [23] using PDA medium. Five mm of mycelia was inoculated on one side of PDA medium plate, while, on the other side, 100 *µ*L of 1 × 10^8^ cell/mL suspension of* D. hansenii* was spread out onto the medium. All plates were incubated for 7 days at 28°C. Three biological replicates from three independent technical replicates were conducted, the fungal colony diameter was measured, and the inhibition of radial growth was calculated as follows:

(1) Inhibition  of  Radial  growth RI%=C−TC×100,

where *C* (control) is the average diameter of fungal colonies in the absence of* D. hansenii* and *T* (treatment) is the average diameter of fungal colonies in cultured plates.

### 2.6. Effect of NaCl, pH, and Temperature on Killer Toxin Production

To detect the optimum condition for the killer toxin production by* D. hansenii*, different factors were examined. Briefly, YEPD was prepared as above and supplemented once with different NaCl concentrations (2, 4, and 8%), or it was either prepared at various pH values 4.6, 5.5, 6.5, and 7 or incubated under different temperatures 15, 20, 25, and 30°C. The* D. hansenii*, under different conditions, was incubated for 72 h under aerobic conditions for 72 h with gentle shaking. The killer toxin activity was evaluated against sensitive yeasts and pathogenic bacterial strains using YEPD-methylene blue agar and Muller Hinton Agar prepared as described above. The clear zones were measured and used as the mean zone of inhibition activity. Yeast growth under different conditions was monitored by measurement of OD_600_ of each condition in comparison to the relevant media as a control (blank). The growth was observed every two hours starting from OD_600_ ≈ 0.1 to approximately OD_600_ ≈ 2.5.

### 2.7. Statistical Analysis

The effect of several growth factors on the killer toxin production and antimicrobial activity was analyzed statistically using One-way ANOVA to determine significant differences in mean values of treated samples as compared with controls. The significance levels were set at *p* < 0.05 using Duncan's range test.

## 3. Results and Discussion

### 3.1. Production of Killer Toxin from* D. hansenii*

**Figure 1:**
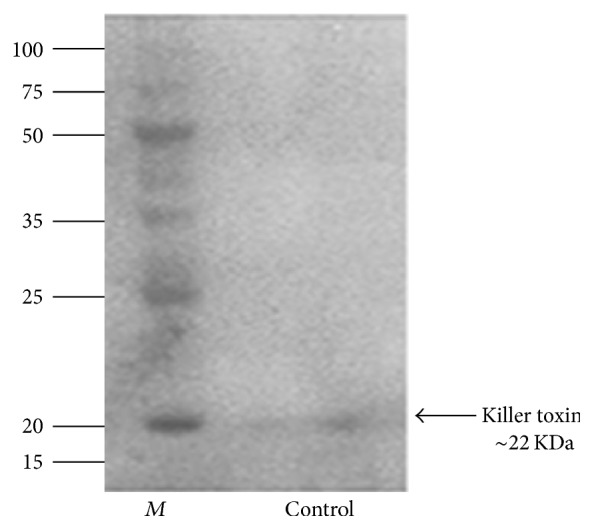
One-dimensional SDS-PAGE of partially purified concentrated killer toxin. 12% SDS-PAGE was performed with the partially purified concentrated killer toxin produced by* D. hansenii.* The 22 kDa protein is referred to by black arrow. Lane* M*: the PageRuler™ Plus Unstained Rec. Protein Ladder (Promega), lane: referring to the purified protein under normal conditions. ~15 *µ*g of protein was loaded into the well.

The* D. hansenii* broth culture was centrifuged and the supernatant was collected for killer toxin isolation. The concentrated killer toxin was subjected and electrophoresed on 12% SDS-PAGE. The result indicates that the molecular weight of the killer toxin was estimated to be 22.000 Da according to the corresponding standard ladder (Figure 1). The present study is focused on the determination of the antimicrobial activity of killer toxin and biological control of* D. hansenii* DSMZ70238 against several pathogens. This yeast is able to produce killer toxin (killer protein) with low molecular protein probably with a 23-kDa portion and encoded by chromosomal genes [13]. From the above result, we found that the* D. hansenii* secreted killer toxin with a molecular weight was about 22 KDa.

**Figure 2:**
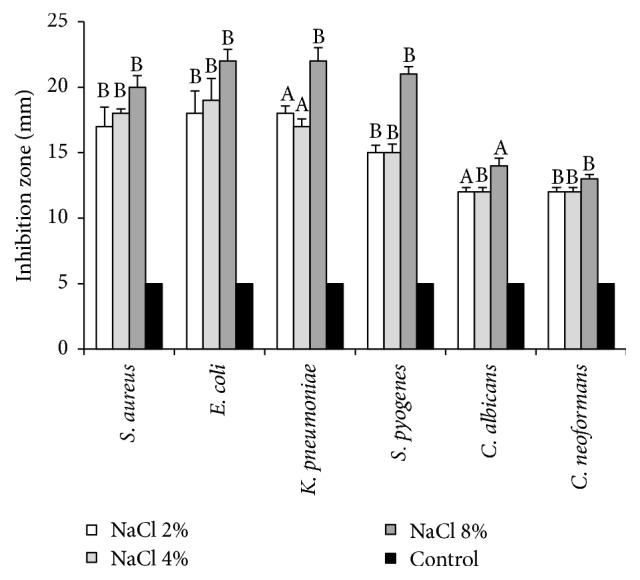
Effect of NaCl concentrations on the production and killing activity of killer toxin produced by* D. hansenii* against pathogenic bacteria and yeasts. Killer activity was measured by the means of inhibition zone around the wells. The shown data are the means and SD from three independent experiments. Values in the same column followed by the same letter are not statistically different by Duncan's multiple range test at (*p* < 0.05).

In order to determine the activity of killer toxin produced by of* D. hansenii*, partially purified and concentrated supernatant was tested using different types of pathogenic microorganisms. The results showed high activity of killer toxin against all tested pathogenic bacteria* S. aureus, E. coli, K. pneumonia, *and* S. pyogenes *and moderate activity against sensitive yeasts* C. albicans and C. neoformans*. The clear zone around wells was measured and considered as an indicator for killer activity. The effect of NaCl concentration on the activity of killer toxin was examined using the agar diffusion well bioassay.  Figure 2 shows the mean diameters of the inhibition zones under different concentrations of NaCl. The optimum inhibitory effect against all pathogens is acquired with 8% NaCl; the clear zone diameters were 20, 22, 22, 21, 14, and 13 mm for* S. aureus, E. coli, K. pneumonia, S. pyogenes C. albicans, *and* C. neoformans,* respectively. The concentration of 2% NaCl showed the lowest antimicrobial effect on the tested pathogenic microbes with inhibition zone diameter from 17 to 12 mm, compared to the control. In addition, Figure 2 shows the effect of NaCl concentration on the activity of killer toxin produced by* D. hansenii* against all pathogens.

**Figure 3:**
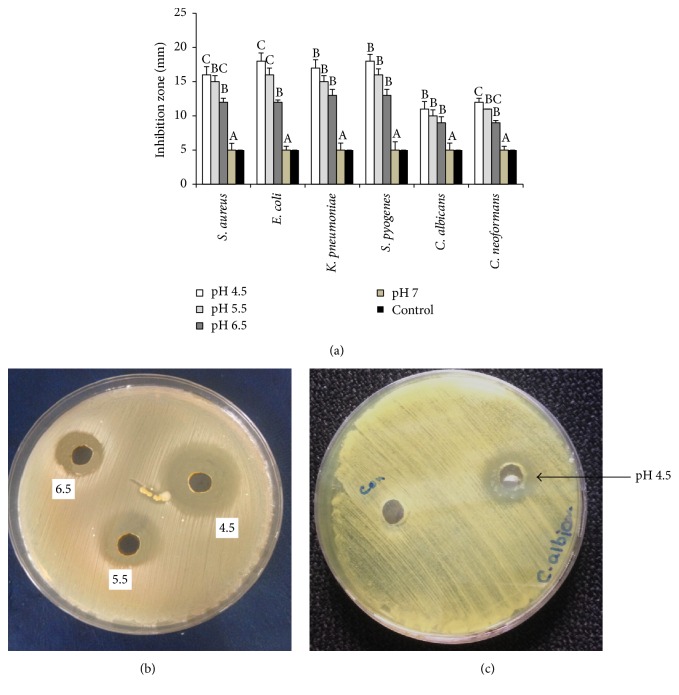
(a) Effect of different pH values on the production and killing activity of killer toxin produced by* D. hansenii* against pathogenic bacteria and yeasts. (b) Inhibition zone distributed within wells filled with partially purified concentrated killer toxin after 48 h at 37°C.* S. aureus* was used as sensitive bacteria. (c)* C. albicans* was used as sensitive yeast. Values in the same column followed by the same letter are not statistically different by Duncan's multiple range test at (*p* < 0.05).

To investigate the effect of pH on the production of killer toxin and its antimicrobial activity, the killer toxin production medium was adjusted with different pH values 4.5, 5.5, 6.5, and 7.  Figure 2 shows the effect of partially purified and concentrated killer toxin on the antimicrobial activity. At optimal pH 4.5, the largest inhibition zones were observed 16, 18, 17, 18, 11, and 12 mm for* S. aureus, E. coli, K. pneumonia, S. pyogenes C. albicans, *and* C. neoformans,* respectively, whereas under the rest of pH values the toxin varied in its ability to reduce the growth of testing pathogens. The results also showed that the neutral medium reduced the killer activity of the concentrated killer toxin, with the lowest inhibition zone observed (5 mm) at pH 7.0 for all tested pathogens. Figures 3(b) and 3(c) show the effect of pH on the killer activity of the concentrated killer toxin against pathogenic* S. aureus* and* C. albicans*. The stability, the activity, and the production of all characterized killer toxins are strongly dependent on the temperature of incubation and the pH values [2, 4, 14]. Moreover, some yeast produce highly active killer toxin under high concentration of salts [21, 24]. In our study, the optimal conditions for killer toxin production from* D. hansenii* DSMZ70238 were investigated; we found that the killer toxin produced by this yeast exhibits more killer activity against pathogenic yeasts and bacteria when the concentration of NaCl is 8%. Previous studies showed that the production, activity, and stability of the killer toxin produced by yeast are increased with the increasing of salt concentration. A study by Hau et al. [24] showed that the addition of 3% NaCl in the production medium gave the highest killer activity of the crude killer toxin produced by* Mrakia frigida* 2E00797 against the pathogenic yeast* Metschnikowia bicuspidata *WCY, while the purified killer toxin showed the highest killer activity when treated with 3.0% of NaCl [25]. The marine yeast* Williopsis saturnus* WC91-2 produced killer toxin that had the maximum killing activity against pathogenic yeast* M. bicuspidate* WCY when the medium was supplemented with 10% NaCl [21]. However, little information is known about the mechanisms that affect the production and killing activity of killer toxin in the presence of NaCl [12].

**Figure 4:**
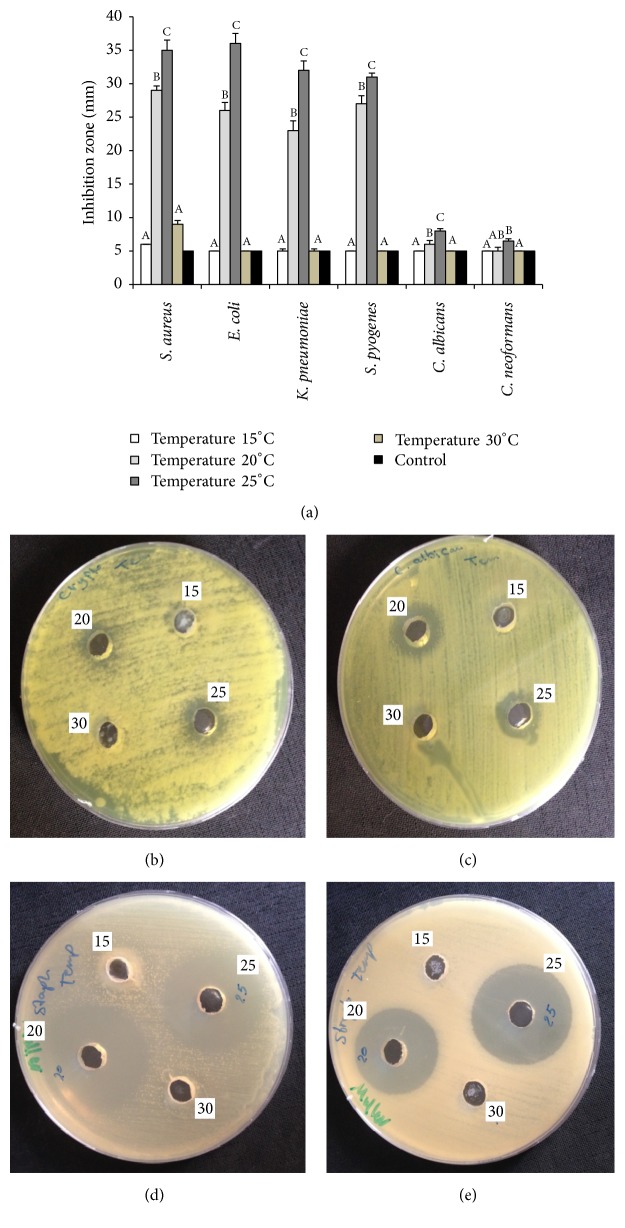
(a) Effect of different temperatures on the production and killing activity of killer toxin produced by* D. hansenii* against pathogenic bacteria and yeasts. Killer activity was measured by the means of inhibition zones around the wells filled with partially purified and concentrated killer toxin. (b)* C. neoformans*, (c)* C. albicans*, (d)* S. pyogenes*, and (e)* S. aureus*. Values in the same column followed by the same letter are not statistically different by Duncan's multiple range test at (*p* < 0.05).

The optimum temperature of the killer toxin activity was determined by incubating the yeast under different temperatures 15, 20, 25, and 30°C. After 72 h incubation time, the results showed that the 25°C is the optimal temperature for toxin killing activity as demonstrated by largest inhibition zones 35, 36, 32, 31, 8, and 6.5 mm for* S. aureus, E. coli, K. pneumonia, S. pyogenes C. albicans, *and* C. neoformans,* respectively.  Figure 4 shows that the smallest inhibition zone was observed at 15°C with value ranging from 6 to 5 mm in diameter. Figures 4(b)–4(e) show the inhibition zones within the wells filled with partially purified and concentrated toxin produced at different temperatures against and* C. albicans* and* C. neoformans, S. aureus, *and* S. pyogenes*.

The ability of killer toxin to inhibit the pathogenic microorganisms was examined under two different conditions. The yeast culture was grown aerobically under temperatures ranging from 20 to 25°C and at pH values ranging from 4 to 4.5. The results showed that the produced killer toxin did not exert any activity against pathogens (data not shown).

Several studies revealed that the killer toxins are well produced and stabilized under low pH values and low temperature [26, 27].* Kluyveromyces siamensis* HN12-1 produced high amount of killer toxin and showed a remarkable activity against the pathogenic yeast* M. bicuspidata* WCY in crab when incubated at 25°C and low pH 4.4–4.0 [28]. In a study performed by Hau et al., [24], the antimicrobial activity of the crude killer toxin was investigated against pathogenic yeast* M. bicuspidata* WCY; the results showed a high killer activity when it grew under acidic condition pH 4.5 and at 15°C, whereas Wang et al. [21] showed that the purified killer toxin produced by the marine yeast* W. saturnus* WC91-2 had high antimicrobial activity against pathogenic yeast* M. bicuspidata* WCY under low value of pH (3–3.5) and at 16°C. The killer activity of killer toxin produced by* D. hansenii* isolated from cheese was investigated against pathogenic* C. albicans* and* C. tropicalis, *and the optimal temperature and pH values were evaluated. The results of this study revealed that the killing activity of killer toxin is mainly affected by the growth conditions like temperature and pH [2].

Our study was performed to assess the activity of killer toxin against Gram (+) and Gram (−) pathogenic bacteria; however, there are fewer studies about the activity of killer toxin against pathogenic bacteria and killer toxin mechanism that mediated the inhibition of Gram (+) bacterial growth [29].

### 3.2. *In Vitro* Antagonistic Activity of* D. hansenii* against Fungal Pathogens

**Figure 5:**
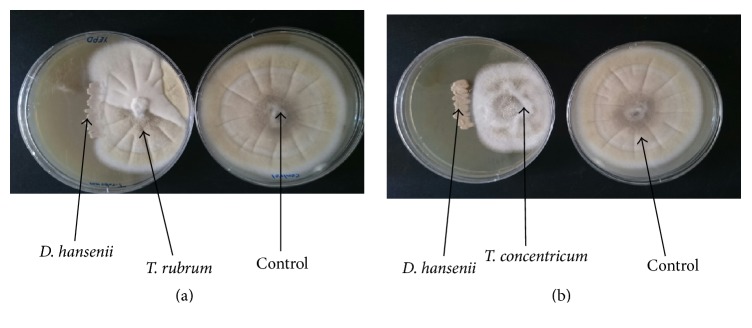
The* antagonistic* activity of* D. hansenii in vitro*. The ability of* D. hansenii *to decrease the growth of* T. rubrum* (a) and* T. concentricum* (b) was examined.* D. hansenii *was streaked out and either* T. rubrum* or* T. concentrcum* was inoculated on the same plate. As a control both* T. rubrum* and* T. concentricum* were inoculated separately without* D. hansenii*. All plates were incubated at 28°C for 7 days. All strains were indicated by black arrows.

Table 1 and Figure 5 show the antagonistic activity of* D. hansenii* against 4 fungal pathogenic strains studied* in vitro*, two human pathogens,* T. rubrum* and* T. concentricum,* and two plant pathogens,* A. alternata *and* C. lunata*. The results showed that* D. hansenii* significantly inhibited the fungal mycelial growth for all tested pathogens and the percentage of inhibition was 47.77, 48.88, 52.22, and 61.11% for* T. rubrum*,* A. alternata, T. concentrcum, *and* C. lunata, *respectively.

The antagonistic ability of* D. hansenii* against pathogenic fungi was previously mentioned by Grzegorczyk et al. [30]. In this study, the* in vitro* and in vivo activity of* D. hansenii *KI2a*, D. hansenii *MI1a, and* Wickerhamomyces anomalus* BS91 was examined against* Monilinia fructigena* and* Monilinia fructicola*. The result showed that the highest* in vitro* killing activity was by* D. hansenii *KI2a and* W. anomalus* BS91, and the mechanisms which mediated their killing activity may be attributed to secreted killer toxins, hydrolytic enzymes production, and production of volatile organic compounds (VOCs). Moreover, the study revealed that the infection of* M. fructigena* and* M. fructicola* on peach and plum was remarkably reduced by both* D. hansenii *KI2a and* W. anomalus* BS91, when they were applied prior to pathogenic inoculation. In addition, no potential action against* M. fructigena* and* M. fructicola* could be observed by* D. hansenii *MI1a.

### 3.3. Growth Curve of* D. hansenii*

**Figure 6:**
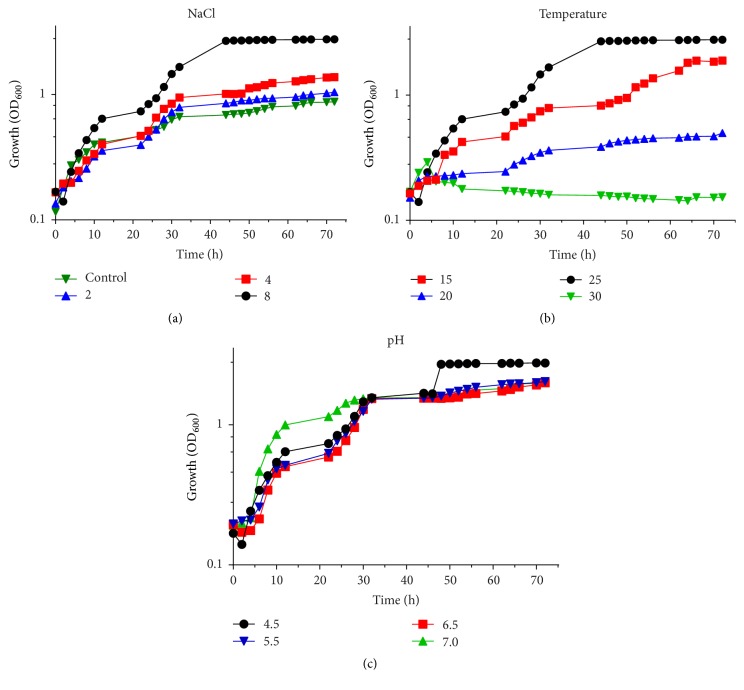
Growth curve of* D. hansenii *DSMZ70238.* D. hansenii *DSMZ70238 was cultured in growth media under different conditions for 72 h. (a) The growth of* D. hansenii *DSMZ70238 with different concentrations of NaCl, (b) the growth under different temperatures, and (c) growth of* D. hansenii* under different pH values.

The growth curve of* D. hansenii* was monitored in culture media supplemented with different NaCl concentrations (2, 4, and 8%). In Figure 6(a), the results showed that the growth rate was enhanced by the addition of different NaCl concentrations. The effect of temperature on the growth curve of this yeast was also studied. The* D. hansenii* culture was incubated under different temperatures 15, 20, 25, and 30°C, and monitored after 72 h. The maximum level of growth rate was observed at 25°C (Figure 6(b)). The results also showed that this yeast is unable to grow at 37°C. The ability of* D. hansenii* to grow in the YEPD medium at different pH values 4.5, 5.5, 6.5, and 7 was determined (Figure 6(c)), and the results indicated that the highest growth was observed at pH 4.5 and that the growth decreased with increasing pH values.

Basically, killer toxin acts mainly on the surface of the yeast cells by special mechanism that includes the interaction with the cell wall components and causes the inhibition of the *β*-glucan synthesis. Alternatively, it may hydrolyse the *β*-glucan in the cell wall of target cells or block the DNA synthesis and cleavage of tRNA. Finally, ion leakage occurs due to the formation of channels in the cytoplasmic membrane and blocking of calcium uptake [10, 19, 31]. Several studies were pointed out on the use of yeasts as biocontrol agents against pathogens like fungal pathogens and using yeast as an alternative solution to synthetic of fungicides [32–34]. In this study, we used this yeast to determine the antifungal activity against four species of fungal pathogens* T. rubrum*,* T. concentricum, A. alternata, *and* C. lunata*. Our results showed that this yeast shows high efficiency in the reduction of radial growth of tested pathogens and the values of antifungal activity ranged between 48.44 and 62.66%. Researchers working in the field of biological control recognized that different yeast species can play important roles in the reduction of disease and prevention of the infection. They can also play a lethal role for pathogens and decrease the colonization of host tissue [32, 35, 36]. Previous study investigated that the zygocin killer yeast* Zygosaccharomyces bailii* has killing activity against human, phytopathogenic yeasts, and filamentous fungus* Fusarium oxysporum* using a standard agar diffusion method [37]. The results showed a significant inhibition of mycelial growth. Moreover,* Pichia membranifaciens* and* Sporobolomyces roseus *were investigated to be good controller for plant pathogenic* Penicillium expansum* and they could be promising alternative biocontrol agents of the growth of this pathogen [38]. The antagonistic activity of yeasts has been attributed to several mechanisms that include (1) competition for nutrients, especially sugars and iron, (2) changes of pH medium through growth-coupled ion exchange or by production of organic acid, (3) tolerance to high concentrations of ethanol [39], and (4) production of antimicrobial compounds, such as killer toxin (mycocin) [40, 41].

## 4. Conclusion

Our results revealed that the* D. hansenii* DSMZ70238 secreted killer toxin, and the antimicrobial activity of killer toxin and its optimizing conditions were studied. The results proved that the optimal killer activity was obtained at high concentration of NaCl, low pH, and mesophilic temperature. Moreover, results showed that this yeast has a good role in the reduction of radial growth of tested pathogenic bacteria and biological control against our studied fungi. Also the growth curve of* D. hansenii* DSMZ70238 was affected by NaCl, pH concentration, and temperature, and the optimal growth conditions were estimated.

## Figures and Tables

**Table 1:** Antifungal activity of *D. hansenii* against tested fungal pathogens measured by the growth inhibition percentage *in vitro*^a^.

Pathogens	Diameter of colony in control/mm	Diameter of colony in treatment/mm	Percentage of inhibition (%)^b^
*T. concentricum*	90	43	52.22 ± 0.23^abc^
*T. rubrum*	90	47	47.77 ± 0.33^b^
*A. alternata*	90	46	48.88 ± 0.30^ab^
*C. lunata*	90	35	61.11 ± 0.15^a^

^a^values are the mean inhibition growth percentage from three replications; ^b^values in the same column followed by the same letter are not statistically different by Duncan's multiple range test at (*p* < 0.05); ^c^± represents standard deviation.
